# A subset of evolutionarily conserved centriolar satellite core components is crucial for sperm flagellum biogenesis

**DOI:** 10.7150/thno.117118

**Published:** 2025-06-12

**Authors:** Bingbing Wu, Chenghong Long, Jiayi Liu, Xiaoming Huang, Shuang Ma, Yanjie Ma, Liying Wang, Yiran Jiang, Bo Yang, Chunxiu Gong, Li Yuan, Yong Zhang, Zhen Li, Wei Li

**Affiliations:** 1 Guangzhou Women and Children's Medical Center, Guangzhou Medical University, Guangzhou, 510623, China.; 2 Department of Human Anatomy, Histology and Embryology, Air Force Medical University, Xi'an, 710032, China.; 3 Department of Endocrinology, Genetics, Metabolism, Beijing Children's Hospital, Capital Medical University, National Center for Children's Health, Beijing, 100045, China.; 4 Beijing Key Laboratory for Genetics of Birth Defects, Beijing Children's Hospital, Capital Medical University, National Center for Children's Health, Beijing, 100045, China.; 5 Savaid Medical School, University of Chinese Academy of Sciences, Beijing, 100049, China.; 6 Key Laboratory of Zoological Systematics and Evolution, Institute of Zoology, Chinese Academy of Sciences, Beijing, 100101, China.; 7 Beijing Hospital, National Center of Gerontology, Institute of Geriatric Medicine, Chinese Academy of Medical Sciences and Peking Union Medical College, Beijing, 100730, China.

**Keywords:** centriolar satellites, CCDC13, PCM1, flagellum biogenesis, male infertility

## Abstract

**Rationale**: Centriolar satellites are non-membranous cytoplasmic granules that cluster around centrosomes, with pericentriolar material 1 (PCM1) serving as the molecular marker for these structures. Although significant progress has been made in understanding their composition, cellular, and organismal functions over the past decades, the tissue-specific roles of centriolar satellite proteins in sperm flagellum biogenesis and male fertility are still not well understood.

**Methods**: We utilize publicly available data and conduct phylogenetic analysis to explore the tissue distribution and conservation of centriole satellite components across flagellated species. Knockout mouse models for *Ccdc13* and *Pcm1* were constructed to investigate their physiological roles. Sperm morphology and functionality were analyzed using immunofluorescence, transmission electron microscopy, and sperm motility analysis. Immunofluorescence, immunoblotting, co-immunoprecipitation, and proteomics analyses were carried out to elucidate the molecular mechanisms by which CCDC13 regulates sperm flagellum biogenesis.

**Results**: We show that most satellite components are expressed in the testis and associated with ciliary function. Comparative analysis of ciliary-related satellite components across 11 flagellated and non-flagellated species revealed six highly conserved satellite proteins in flagellated species. PCM1, a well-known centriolar satellite scaffolding protein, was found to be less conserved. Based on these findings, we selected CCDC13, a highly conserved satellite protein, and PCM1, a less conserved component, for functional comparison in sperm flagellum biogenesis. Using knockout mouse models, we demonstrated that *Ccdc13* deficiency led to male infertility with multiple morphological abnormalities of the sperm flagella (MMAF)-like phenotype due to defects in sperm flagellum biogenesis. While *Pcm1* knockout only resulted in decreased sperm motility without affecting flagellum biogenesis. Molecularly, CCDC13 interacts with IMT, IFT-associated proteins, and flagellar components to regulate transport of cargo to proper positions for flagellum biogenesis.

**Conclusion**: This study identifies a subset of highly conserved centriolar satellite proteins essential for sperm flagellum biogenesis. The identification of these proteins provides valuable insights into the genetic mechanisms underlying flagellum function and their evolutionary development. Additionally, defects in these proteins may be associated with male infertility in humans.

## Introduction

Cilia are long appendages extending from the cell body and are generally classified into two types in vertebrates based on their function and axonemal structure: non-motile and motile cilia 1-3. Non-motile or primary cilia, typically present as a single structure per cell, primarily function in signal transduction between the extracellular environment and the cell nucleus 3. Motile cilia are characterized by a “9 + 2” microtubule arrangement and serve various functions, including generating fluid flow and facilitating locomotion 4. In some cases, cilia are referred to as flagella when they occur singly or in small numbers and are involved in cell motility, particularly when propelling the cell, as in the case of the sperm flagellum 5. The sperm flagellum is a highly specialized motile cilium, significantly longer than other cilia and containing sperm-specific structures that are essential for swimming and fertilization 4.

All types of cilia emanate from the basal body derived from the mother centriole and protrude from the plasma membrane. The basal body is crucial for assembling the transition zone and axoneme in conjunction with intraflagellar transport (IFT), which acts as an intracellular cargo delivery system, akin to the train system 6,7. IFT is responsible for transporting preassembled axonemal components from the base of the cilium to its tip, as well as disassembled components in the opposite direction 5. Sperm flagellum axoneme formation resembles motile cilia assembly with testis-specific isoforms. It extends from the sperm basal body (distal centriole), which is anchored to the implantation fossa of the nucleus and serves as the core structure along the flagellum 4. Unlike the formation of other motile cilia, the assembly and transportation of sperm flagellum structural proteins during spermatid elongation require both intramanchette transport (IMT) and intraflagellar transport (IFT) to reach the developing head and tail. The manchette is a transient, skirt-like structure that surrounds the elongating spermatid head and exists solely during spermatid elongation 8. Defects in sperm flagellum biogenesis result in various flagellar abnormalities, such as absence, bending, coiling, shortening, and irregularity, which are collectively referred to as multiple morphological abnormalities of the sperm flagella (MMAF). MMAF is a distinct form of asthenoteratozoospermia, defined by a significant genetic basis 9,10.

Numerous proteins participate in the biogenesis and maintenance of cilia/flagella through dynamic interactions with multimeric protein complexes localized at the basal body, centrosome, and centriolar satellites 11,12. Centriolar satellites are 70- to 100-nm non-membranous granules that cluster around the centrosome/cilium complex in a manner dependent on microtubules and dynein 13,14. Although first described in the early 1960s, it was not until decades later that PCM1 was identified as the first satellite protein 15. Satellite components were subsequently identified based on their co-localization with and/or interaction with PCM1 in the pericentriolar area surrounding the centrosome. Currently, over 65 proteins have been defined as centriolar satellite proteins 16. Recent evidence has demonstrated the essential regulatory role of centriolar satellites in the formation of the centrosome/cilium complex. During ciliogenesis, centriolar satellites serve as platforms for assembling ciliary components and facilitate their transport to the growing cilium 17,18. Centriolar satellites play a crucial role in recruiting proteins, vesicles, and signaling molecules that are essential for cilia formation and function. Although significant progress has been made in understanding the composition and cellular/organismal functions of centriolar satellites 12,16,19-21, the tissue-specific functions of centriolar satellite proteins and their relationship with sperm flagellum biogenesis and male fertility are still not well understood.

Here, we found that most centriolar satellite components are expressed in the testis and are associated with ciliary function. Phylogenetic analysis of the ciliary-related satellite proteins further highlighted six highly conserved satellite components (BBS4, DZIP1, CCDC113, CCDC13, CEP131, and TEX9) in flagellated species. The knockout of the *Ccdc13* gene led to hydrocephalus and male infertility, accompanied by severe defects in the formation of ependymal cilia and sperm flagella. In contrast, deficiency of the less conserved satellite protein PCM1 only resulted in asthenospermia and partial sperm head deformity but did not affect sperm flagellum biogenesis. Further studies have revealed that CCDC13 functions as a cargo protein, interacting with IFT-B components and the kinesin-2 motor subunit KIF3A to direct cargo to the developing flagellum. In the absence of CCDC13, the transport of HOOK1 and ODF2 was disrupted. This disruption subsequently led to abnormal elongation of the manchette and disordered cargo transport within the flagellum, ultimately affecting sperm flagellum formation. Together, our results reveal a set of conserved satellite proteins that are essential for sperm flagellum biogenesis. The identification of these proteins provides valuable insights into the genetic basis of sperm flagellum biogenesis, suggesting that defects in these proteins might be associated with male infertility in humans.

## Materials and Methods

### Animals

The mouse *Ccdc13* gene (Transcript: ENSMUSG00000079235) is located on chromosome 9 and spans 61.84 kb, consisting of 16 exons. Exons 3 to 10 were selected as the target region.* Ccdc13^-^*knockout founder mice were generated by Cyagen Biosciences using the CRISPR-Cas9 system. The Cas9 mRNA and gRNA were co-injected into fertilized C57BL/6J mouse eggs to produce heterozygous mice with an 18096 base pair (bp) deletion. These heterozygotes were mated to produce offspring, which were then genotyped using specific primers to identify *Ccdc13^+/+^* and *Ccdc13^-/-^*mice. Detailed primer information is provided in Table S1.

The Institute of Cancer Research (ICR) genetic background was introduced by crossing C57BL/6J heterozygous mice with ICR wild-type mice. The resulting heterozygotes were further interbred to generate *Ccdc13^-/-^* mice in the mixed C57BL/6J (ICR) background. Male mice of different genotypes at 2 months of age were subjected to fertility testing and phenotypic analysis.

The mouse *Pcm1* gene (Transcript: ENSMUSG00000031592) is located on chromosome 8 and spans 112.59 kb, consisting of 38 exons. Exons 3 to 6 were selected as the target region. *Pcm1^-^*knockout founder mice were generated by Cyagen Biosciences using the CRISPR-Cas9 system. The Cas9 mRNA and gRNA were co-injected into fertilized C57BL/6N mouse eggs to produce heterozygous mice with a 10422 bp deletion. These heterozygotes were mated to obtain offspring, which were then genotyped using specific primers to identify *Pcm1^+/+^* and *Pcm1^-/-^*mice. Detailed primer information is provided in Table S1.

Ethical approval for all animal experimental procedures was provided by the Laboratory Animal Welfare and Ethics Committee of Guangzhou Women and Children's Medical Center (Ethics No. RSDW-2024-01391).

### Assessment of fertility

The fertility of 2-month-old male mice with different genotypes was evaluated by mating each male with three 6- to 8-week-old WT C57BL/6 females. Vaginal plugs were checked daily, and females with plugs were subsequently isolated and housed individually. The birth dates and litter sizes were recorded. Females that failed to deliver by day 21 post-coitus were classified as non-pregnant and euthanized for confirmation.

### Identification of centriolar satellite protein homologues in different organisms

Amino acid sequences of the centriolar satellite proteins were queried against human homologues, which were retrieved from UniProt and NCBI. Protein homologues were identified using a BLASTP search through NCBI, ENSEMBL, and UniProt, applying a stringent e-value threshold of 10^-5^ or lower. Additionally, the InterPro online tool (http://www.ebi.ac.uk/interpro/) was used to further examine these potential homologous proteins.

### Protein-protein interaction (PPI) network and tissue-specific gene enrichment analysis

PPI network analysis and Gene Ontology (GO) enrichment of satellite proteins were performed using STRING v12 (https://cn.string-db.org/) and Gene Ontology (http://www.geneontology.org). The PPI network between CCDC13 and its interactors was analyzed using the STRING database and Cytoscape software (version 3.10.3). We performed tissue-specific gene enrichment analysis using the TissueEnrich R package, based on RNA-Seq data from the Human Protein Atlas and Mouse ENCODE.

### Immunoprecipitation (IP)

Testicular tissues were lysed in IP lysis buffer containing 150 mM NaCl, 20 mM HEPES, 1 mM DTT, 0.5% NP-40, pH 7.3, with a protease inhibitor cocktail (Roche Diagnostics, 04693132001) added on ice. The lysates were cleared by centrifugation at 12,000 × g for 25 min at 4 °C, followed by overnight incubation with antibodies on a shaker at 4 °C. Afterward, the supernatants were incubated with protein A/G-coated agarose beads at 4 °C for 3 h, washed 4 times with lysis buffer, and prepared for subsequent western blot or liquid chromatography-tandem mass spectrometry (LC-MS) analysis.

### Proteomics analysis

Gel pieces were destained with 50% acetonitrile, dehydrated in 100% acetonitrile, and then digested overnight with trypsin at 37 °C. The resulting peptides were extracted, dried, resuspended in 2% acetonitrile/0.1% formic acid, and desalted using StageTip. Tryptic peptides were separated using an EASY-nLC 1200 UPLC system and analyzed with an Orbitrap Exploris 480 mass spectrometer, coupled with a nano-electrospray ion source. Full MS scans were performed at a resolution of 60,000 (350-1800 m/z), and MS/MS was conducted at a resolution of 15,000 with TurboTMT turned off. The 20 most abundant precursors were chosen for MS/MS analysis, with a 20 s dynamic exclusion. HCD fragmentation was carried out at 28% NCE, and the AGC target was set to 50%, with an intensity threshold of 5,000 ions/s. Data processing was performed using the PD search engine (v.2.4) against the Mus_musculus_10090_SP_20231220_add.fasta database. Trypsin/P was specified as the enzyme, with allowance for up to 2 missed cleavages. Modifications included carbamidomethylation on Cys (fixed), acetylation on N-terminal, and oxidation on Met (variable). Peptides with a score >20 and high confidence were considered for identification.

### Immunoblotting

Tissue protein extracts were prepared by incubating tissues in lysis buffer containing a protease inhibitor cocktail. The solution was briefly sonicated and incubated on ice for 40 minutes. The samples were centrifuged at 12,000 rpm for 25 min at 4°C, and the supernatant was carefully collected. Protein lysates were loaded onto a gel for electrophoresis and then transferred to a nitrocellulose membrane by electroblotting. After blocking the membrane with 5% skim milk, it was incubated with primary and secondary antibodies. The membrane was subsequently scanned using an Odyssey infrared imager (LI-COR Biosciences, RRID: SCR_014579). Antibody details are provided in Table S2.

### Sperm count

Sperm count was performed by isolating the cauda epididymis from 2-month-old mice, releasing spermatozoa with phosphate-buffered saline (PBS), incubating at 37 °C for 10 min, and then counting the sperm using a hemocytometer.

### Sperm motility assessment

Spermatozoa were extracted from the unilateral cauda epididymis of 2-month-old mice, incubated in 1 mL of PBS at 37 °C for 10 min, and then analyzed for motility. The sperm concentration after dilution varied slightly among samples but was typically around 7 × 10⁶ sperm/mL. After incubation, a 10 μL aliquot of the sperm suspension was collected from each sample and examined under a 20× phase objective (Zeiss) using a Axiolab5 microscope (Zeiss). Viewing areas in each chamber were imaged using a CCD camera (Hamilton Thorne). Motility parameters, including total and progressive sperm motility, were assessed using computer-assisted semen analysis (CASA) with CEROS motility analysis software.

### Transmission electron microscopy (TEM)

The cauda epididymis was carefully dissected and fixed overnight at 4 °C in 2.5% glutaraldehyde dissolved in 0.1 M cacodylate buffer. Following washing in the same buffer, the tissue was sectioned into 1 mm³ pieces and incubated in 1% OsO4 for 1 h at 4 °C. The samples were then dehydrated in an increasing gradient of acetone (50%, 60%, 70%, 80%, 90%, 95%, 100%) and subsequently embedded in resin for further processing. Ultrathin sections were prepared, stained with lead citrate and uranyl acetate, and then analyzed under a TEM (JEM-1400) for image acquisition.

### Immunofluorescence

The testis was rapidly dissected and placed in 2% paraformaldehyde in 0.05% PBST for 5 min. The fixed tissue was then transferred to a slide, gently compressed under a coverslip, and flash frozen in liquid nitrogen. After removing the coverslip, the slides were washed three times with PBS, treated with 0.1% Triton X-100 for 15 min, washed again with PBS, and blocked with 5% bovine serum albumin. The primary antibody was incubated overnight at 4 °C, followed by incubation with the secondary antibody, and the nuclei were subsequently stained with DAPI. Images were captured using a Nikon AXR microscope (Tokyo, Japan) or an SP8 microscope (Leica). Antibody details are provided in Table S2.

### Statistical analysis

All experiments were conducted at least three times, and the results are expressed as the mean ± SD. Statistical comparisons between the mean values of different genotypes were performed using paired, two-tailed Student's t-tests. A *P*-value of less than 0.05(*), 0.01(**), 0.001(***) or 0.0001(****) was considered statistically significant.

## Results

### High expression of most satellite components in the testis, potentially related to cilia

**Figure 1:**
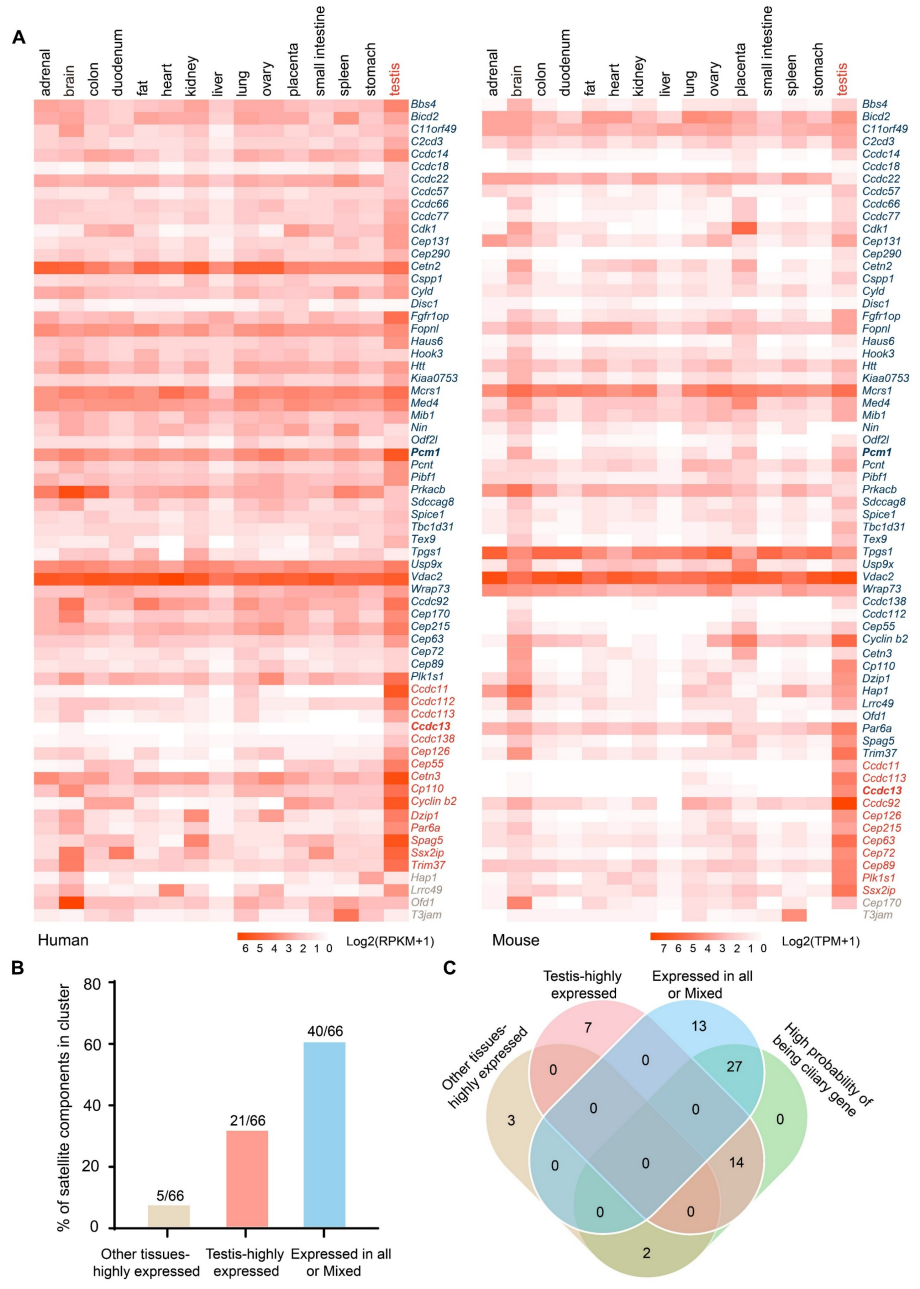
** Expression of most satellite components in testis.** (A) Expression heatmaps for 66 centriolar satellite components in human and mouse tissues were generated using publicly available data: the Human Protein Atlas RNA-seq dataset for human tissues and Mouse ENCODE transcriptome data from NCBI. Most centriolar satellite components were detectable in the testis. TissueEnrich was used to categorize genes into three classes: the “Expressed in all or Mixed”, highlighted in blue; the “Testis-highly expressed”, highlighted in red; and the “Other tissues-highly expressed”, highlighted in brown. (B) The percentage of satellite components in different categories based on TissueEnrich analysis. (C) The Venn diagram compares the cilium-related satellite components predicted by the Ciliogenics database with the three classes of satellite components based on their expression characteristics.

To investigate the biological functions of centriolar satellite components, we examined their expression patterns across different tissues to assess their potential roles in these tissues. Expression heatmaps for 66 centriolar satellite components in human and mouse tissues were generated based on the Human Protein Atlas RNA-seq dataset for human tissues 22 and Mouse ENCODE transcriptome data 23 from the National Center for Biotechnology Information (Figure 1A and Table S3). Our analysis revealed that most centriolar satellite components were detectable in the testis. To characterize the tissue distribution of the satellite proteins, we utilized TissueEnrich 24 and identified three main distribution classes. The first class, defined as “Expressed in all or Mixed”, consists of satellite components that are broadly expressed across various tissues and is highlighted in blue. The second class, defined as “Testis-highly expressed”, consists of satellite components that are highly expressed in testis tissue and is highlighted in red. The third class, defined as “Other tissues-highly expressed”, consists of satellite components that are highly expressed in other tissues and is highlighted in brown. Among them, *Cep170* and* Ofd1* are highly expressed in the brain, *Hap1* is highly expressed in the stomach, *Lrrc49* is highly expressed in the heart, and *T3jam* is highly expressed in the spleen (Figure 1A-B). Further analysis showed that the expression distribution of satellite components in human and mouse tissues was similar, with 40 satellite components identified in the “Expressed in all or Mixed” classes (Figure 1A-B, S1A). It is noteworthy that 21 satellite components were highly expressed in testis tissues in both human and mouse. Among these, five satellite components (*Ccdc11*,* Ccdc113*, *Ccdc13*, *Cep126*, and* Ssx2ip*) were significantly expressed in testis tissue in both humans and mice (Figure 1A-B, S1B).

Given that centriolar satellites localize to and move around centrosomes and cilia, they are essential for regulating ciliary function 19. We used the CilioGenics database 25, which integrates multiple independent methods, to predict potential cilia-related genes among these satellite components. 43 satellite components were predicted to have a high probability of being ciliary genes (Figure 1C, S1C). Notably, 14 out of 21 satellite components that are highly expressed in the testis are cilia-related, suggesting their potential involvement in sperm flagellum function.

### Highly conserved satellite proteins that are essential for sperm flagellum biogenesis were identified

**Figure 2:**
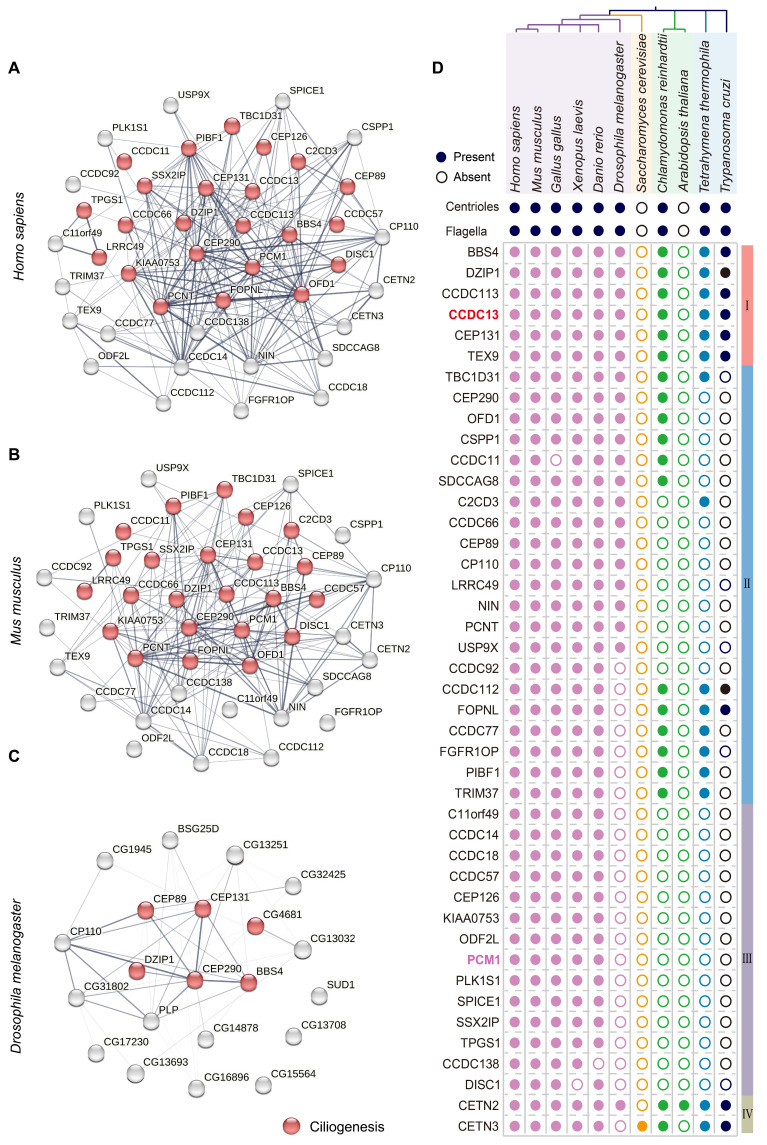
** A set of highly conserved centriolar satellite proteins might be required for sperm flagellum biogenesis.** (A-C) Gene ontology analysis, along with protein-protein interaction networks, was conducted across *Homo sapiens*, *Mus musculus*, and *Drosophila melanogaster* using the STRING database. Satellite proteins involved in ciliogenesis (GO:0060271) are marked by red circles. The thickness of the lines represents the level of support provided by the data. (D) The presence or absence of centrioles and flagella is depicted for 11 organisms representing major eukaryotic lineages in different colors. (Holozoa: violet, Fungi: orange, Plantae: green, Protozoa: blue). Branch lengths are meaningless. Organisms with centrioles and flagella include *Homo sapiens*, *Mus musculus*, *Gallus gallus*, *Xenopus laevis*, *Danio rerio*, *Drosophila melanogaster*, *Chlamydomonas reinhardtii*, *Tetrahymena thermophila*, *Trypanosoma cruzi*. Organisms without centrioles and flagella include *Saccharomyces cerevisiae* and *Arabidopsis thaliana*. Protein homologues of satellite components were identified using a BLASTP search in ENSEMBL, NCBI, and UniProt. In all instances, the query sequences were human homologs of these proteins. Based on their distribution patterns across 11 organisms, satellite proteins were classified into four groups and indicated in the right column: Group I—satellite protein orthologues highly conserved in flagellated organisms but absent in non-flagellated ones; Group II—satellite protein orthologues present to varying extents in some flagellated organisms but absent in non-flagellated ones; Group III—satellite protein orthologues less conserved across flagellated species; and Group IV—satellite protein orthologues present in both flagellated and non-flagellated species.

To identify potential centriolar satellite components involved in flagellum biogenesis, we first constructed protein-protein interaction (PPI) networks for the 43 cilia-related satellite components in *Homo sapiens*, *Mus musculus*, and *Drosophila melanogaster*. Gene Ontology (GO) analysis and literature review revealed that 24 satellite proteins are associated with ciliogenesis and indicated with red circles (Figure 2A-C). Notably, the interaction networks progressively simplified and became more distinct from *Homo sapiens* to *Drosophila melanogaster*, suggesting an evolutionary conservation of certain satellite proteins across species. To investigate the relationship between centriolar satellite proteins and flagella, we selected 11 organisms from four major eukaryotic groups (Holozoa, Fungi, Plantae, and Protozoa), including those with flagella (e.g., *Homo sapiens*, *Mus musculus*, *Gallus gallus*, *Xenopus laevis*, *Danio rerio*, *Drosophila melanogaster*, *Chlamydomonas reinhardtii*, *Tetrahymena thermophila*, and *Trypanosoma cruzii*) as well as those without flagella (e.g., *Saccharomyces cerevisiae* and *Arabidopsis thaliana*). In organisms without flagella and basal bodies, we speculated that they might also lack genes required for building and regulating these organelles 26. Furthermore, we postulated that satellite proteins crucial for flagellum biogenesis would co-distribute with flagella across the eukaryotic phylogenetic tree. We then analyzed the phylogenetic distribution of satellite proteins, correlating their patterns with the presence or absence of flagella. A BLASTP analysis was performed to identify homologous satellite proteins from other species by querying the amino acid sequences of human satellite proteins (Table S4). Based on the distribution patterns of these proteins across 11 organisms, we classified them into four groups as indicated in the right column (Figure 2D). Group I includes BBS4, DZIP1, CCDC113, CCDC13, CEP131, and TEX9, which are the most conserved centriolar proteins across all flagellated organisms but absent in non-flagellated ones. Group II comprises satellite protein homologues present to varying extents in some flagellated species but absent in non-flagellated species. Group III contains satellite proteins that are primarily found in vertebrates and show less conservation among flagellated species. Group IV includes satellite protein homologues present in both flagellated and non-flagellated species, indicating a broader conservation across eukaryotes.

We hypothesize that the highly conserved satellite components in flagellated species may play crucial, conserved roles in both ciliogenesis and flagellum biogenesis, as these components likely retain fundamental functions and may have been among the first proteins involved in forming these structures. Consistent with this hypothesis, a literature search revealed that BBS4 27-31, CEP131 32-35, and DZIP1 36-40 have been reported to play conserved roles in ciliogenesis and sperm flagellum biogenesis. TEX9 has recently been functionally predicted to play a crucial role in ciliogenesis 41,42 and was found to localize to the basal bodies of spermatids in *Drosophila melanogaster*. Disruption of TEX9 resulted in the absence of the inner pair of microtubules in sperm 43. CCDC113 was originally identified as a satellite protein involved in primary cilia formation 44, and our recent work shows that deficiency in CCDC113 lead to abnormalities in sperm flagella 45. Similarly, CCDC13 localizes to the basal body and has been reported to be necessary for primary cilia formation. Depletion of *Ccdc13* resulted in decreased localization of BBS4 to both primary cilium and centriolar satellites in retinal pigment epithelial (RPE1) cells 46. Given that *Ccdc13* is predominantly expressed in the testis of both humans and mice (Figure 1A), it might also be involved in sperm flagellum biogenesis. In addition to CCDC13, we selected PCM1 for further studies because it is not only the first satellite protein identified and a marker of centriolar satellites 13, 20, but also less conserved in flagellated species (Figure 2D). PCM1 has been reported to play a crucial role in ciliogenesis 47-49, but it is unclear whether PCM1 also plays a role in sperm flagellum biogenesis.

### *Ccdc13^-/-^* mice, but not *Pcm1^-/-^* mice, showed sperm flagellum defects

**Figure 3:**
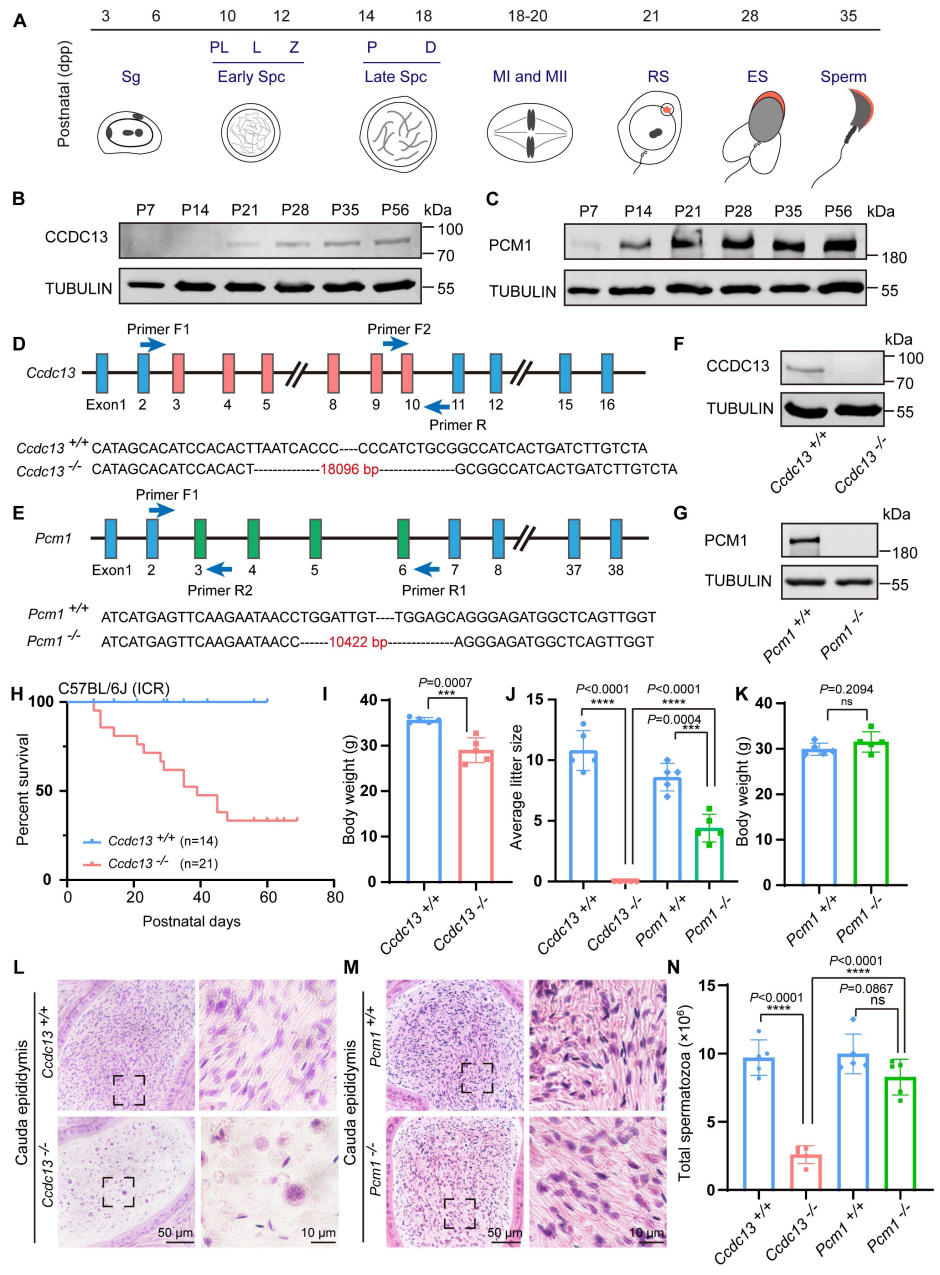
***Ccdc13* knockout leads to male infertility, while *Pcm1* knockout leads to male subfertility.** (A) The different germ-cell stages of spermatogenesis in mice after birth are described. Sg: spermatogonia, Spc: spermatocyte, M, meiotic, RS: round spermatid, ES: elongating spermatid. (B) CCDC13 was expressed starting in P21 testes. TUBULIN served as the loading control. (C) PCM1 was expressed starting in P7 testes. TUBULIN served as the loading control. (D) Generation of *Ccdc13*-knockout mice lacking exon 3 to exon 10. (E) Generation of *Pcm1*-knockout mice lacking exon 3 to exon 6. (F) Immunoblotting of CCDC13 in *Ccdc13^+/+^* and *Ccdc13^-/-^* testes. TUBULIN served as the control. (G) Immunoblotting of PCM1 in *Pcm1^+/+^* and *Pcm1^-/-^* testes. TUBULIN served as the control. (H) Survival rate of postnatal *Ccdc13^-/-^* mice with a C57BL/6J and Institute of Cancer Research (ICR) crossbred background. (I) The body weight of *Ccdc13^-/-^* male mice was reduced compared to those of* Ccdc13^+/+^* male mice (n = 5 independent experiments). Data are presented as the mean ± SD. ***P < 0.001. (J) The average litter size of *Ccdc13^+/+^*, *Ccdc13^-/-^*, *Pcm1^+/+^* and *Pcm1^-/-^* male mice (n = 5 independent experiments). Data are presented as mean ± SD. ***P < 0.001, ****P < 0.0001. (K) The body weight of *Pcm1^+/+^* and *Pcm1^-/-^* male mice (n = 5 independent experiments). Data are presented as mean ± SD. ns: indicates no difference. (L and M) H&E staining of the caudal epididymis from* Ccdc13^+/+^*, *Ccdc13^-/-^*, *Pcm1^+/+^* and *Pcm1^-/-^* male mice. (N) Analysis of sperm counts in *Ccdc13^+/+^*, *Ccdc13^-/-^*, *Pcm1^+/+^* and *Pcm1^-/-^* male mice. (n = 5 independent experiments). Data are presented as the mean ± SD. ****P < 0.0001. ns: indicates no difference.

To explore the potential functions of CCDC13 and PCM1 in the testes, we examined their expression patterns during the first wave of spermatogenesis (Figure 3A). CCDC13 expression was first detected at postnatal day 21 (P21) and gradually increased from P28 onward (Figure 3B), coinciding with the initiation of sperm flagellum formation 50. In contrast, PCM1 showed weak expression at postnatal day 7 (P7), which significantly increased from P14 onward (Figure 3C). The differences in their expression patterns suggest that CCDC13 and PCM1 play distinct roles during spermatogenesis. To investigate the physiological roles of CCDC13 and PCM1, we utilized CRISPR/Cas9 technology to generate *Ccdc13^-/-^* and *Pcm1^-/-^* mice, targeting exons 3 to 10 of *Ccdc13* and exons 3 to 6 of *Pcm1*, respectively (Figure 3D-E).Successful gene knockout was confirmed by genomic DNA PCR (Figure S2A-B) and immunoblotting analysis, which verified the absence of CCDC13 and PCM1 in testis protein extracts from respective knockout mice (Figure 3F-G). *Ccdc13^-/-^* mice in the C57BL/6J background exhibited severe hydrocephalus and were markedly smaller than their littermate controls, resulting in lethality within 10 days after birth (Figure S2C-D). Furthermore, we observed that the formation of ependymal cilia in *Ccdc13^-/-^* mice was significantly affected (Figure S2E), suggesting that CCDC13 is crucial for ciliogenesis in ependymal cells. Given that homozygous knockout mice in the C57BL/6J background could not survive to adulthood, we introduced the Institute of Cancer Research (ICR) genetic background by crossing *Ccdc13^+/-^* (C57BL/6J) mice with ICR mice. This strain is known for its robust health, strong adaptability, and good reproductive performance 51. The resulting heterozygotes were further interbred to generate *Ccdc13^-/-^* mice in the mixed C57BL/6J (ICR) background. Notably, approximately 33% (7/21) of these *Ccdc13^-/-^* mice survived beyond 2 months (Figure 3H). The body weight of 2-month-old *Ccdc13^-/-^* male mice was reduced compared with the controls (Figure 3I). Fertility analysis of 2-month-old male *Ccdc13^-/-^* mice in the C57BL/6J (ICR) background revealed that, although they displayed normal mating behavior as indicated by the presence of copulatory plugs, they were unable to sire offspring when paired with WT female mice (Figure 3J). In contrast, *Pcm1^-/-^* male mice showed no apparent abnormalities in appearance or behavior, and their body weight did not significantly differ from that of *Pcm1^+/+^* male mice (Figure 3K). However, fertility analysis revealed that 2-month-old male *Pcm1^-/-^* mice in the C57BL/6N background were subfertile. The average litter size of *Pcm1^+/+^* male mice (8.6±1.14) was significantly higher than that of *Pcm1^-/-^* male mice (4.4±1.14) (Figure 3J).

**Figure 4:**
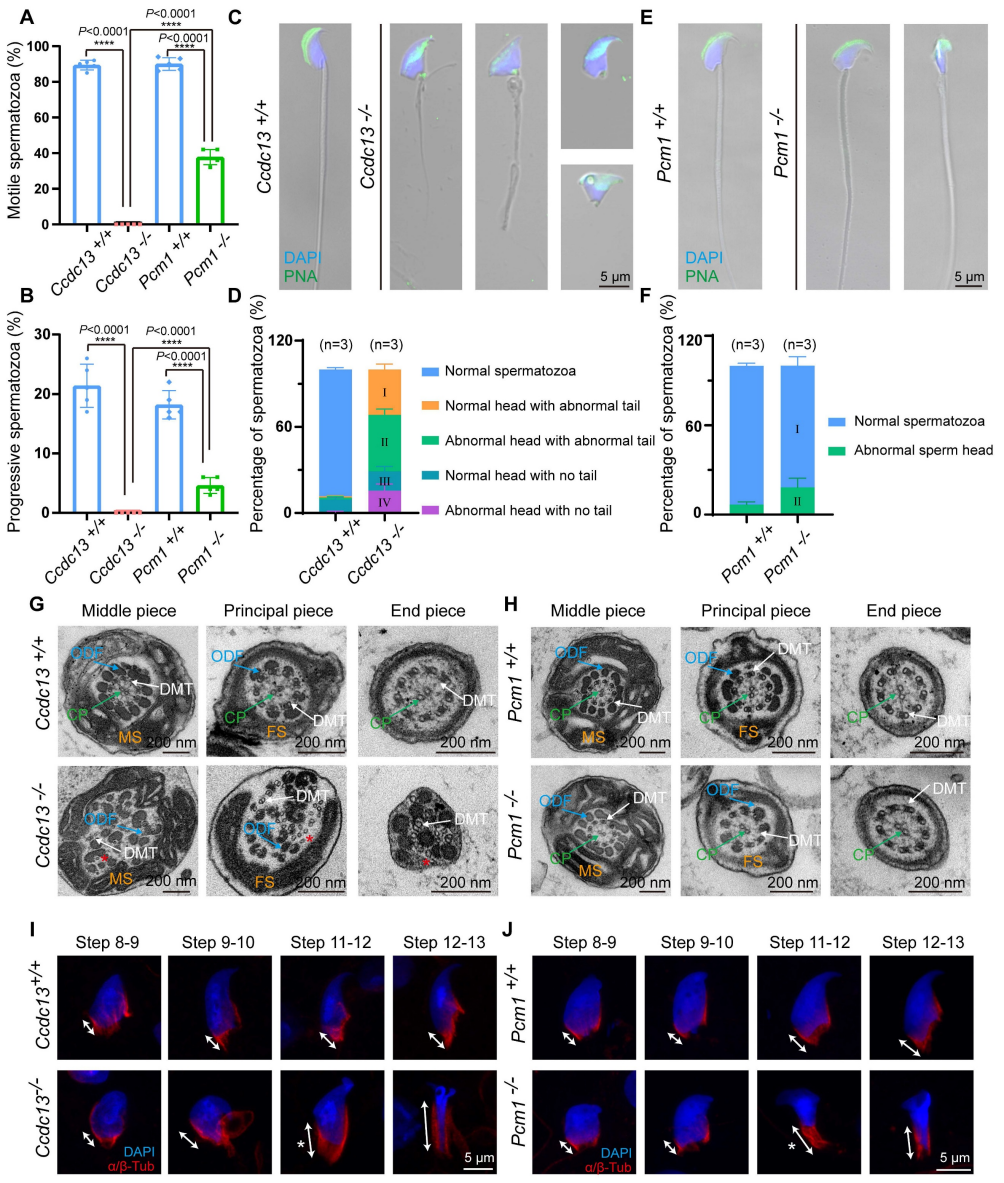
***Ccdc13* knockout results in sperm flagellum defects, while *Pcm1* knockout does not affect flagellum structure.** (A and B) Analyses of sperm motility, progressive motility of *Ccdc13^+/+^*, *Ccdc13^-/-^*, *Pcm1^+/+^* and *Pcm1^-/-^* male mice (n = 5 independent experiments). Data are presented as the mean ± SD. ****P < 0.0001. (C) Fluorescence staining of PNA in *Ccdc13^+/+^*, *Ccdc13^-/-^* spermatozoa. (D) Quantification of different categories of *Ccdc13^+/+^*, *Ccdc13^-/-^* spermatozoa (n = 3 independent experiments). Data are presented as the mean ± SD. (E) Fluorescence staining of PNA in *Pcm1^+/+^* and *Pcm1^-/-^* spermatozoa. (F) Quantification of different categories of *Pcm1^+/+^* and *Pcm1^-/-^* spermatozoa (n = 3 independent experiments). Data are presented as the mean ± SD. (G and H) TEM analysis of spermatozoa from the cauda epididymis of *Ccdc13^+/+^*, *Ccdc13^-/-^*, *Pcm1^+/+^* and *Pcm1^-/-^* male mice. MS: mitochondrial sheath, FS: fibrous sheath, ODF: outer dense fibers, DMT: doublet microtubule, CP: central pair. Red asterisks indicate the disintegrated DMT. (I and J) Spermatids at various stages containing the manchette were stained with antibodies against α/β-tubulin (red) to visualize the manchette (double headed arrows) in *Ccdc13^+/+^*, *Ccdc13^-/-^*, *Pcm1^+/+^*, and *Pcm1^-/-^* male mice. White asterisks indicate the abnormally elongating manchette.

To explore how knockout of *Ccdc13* and *Pcm1* influences male fertility, we histologically examined the *Ccdc13^-/-^* and *Pcm1^-/-^* cauda epididymis. H&E staining revealed fewer spermatozoa in the epididymal lumen of* Ccdc13^-/-^* mice compared to *Ccdc13^+/+^* mice (Figure 3L). However, no significant difference in sperm count was observed between *Pcm1^-/-^* and *Pcm1^+/+^* mice (Figure 3M). Further sperm analysis showed a significant decrease in the number of spermatozoa released from the cauda epididymis of *Ccdc13^-/-^* mice (Figure 3N), with sperm appearing 100% immotile, in contrast to the motile sperm of *Ccdc13^+/+^* mice (Figure 4A). In *Pcm1^-/-^* mice, sperm count in the cauda epididymis was similar to that of *Pcm1^+/+^* mice (Figure 3N), but sperm motility was markedly reduced (Figure 4-B). *Pcm1^+/+^* mice produced 90.00±3.53% motile sperm, including 18.20±2.38% progressive sperm. *Pcm1^-/-^* mice produced only 37.80±4.26% motile sperm, including 4.60±1.34% progressive sperm (Figure 4-B).

To examine the morphological features of *Ccdc13^-/-^* and *Pcm1^-/-^* spermatozoa, we used fluorescence staining with lectin peanut agglutinin (PNA) to visualize the acrosome of spermatozoa. *Ccdc13^-/-^* spermatozoa exhibited morphological abnormalities, including short, curly or absent flagella and non-sickle-shaped abnormal nuclei (Figure 4C). Figure 4D shows the percentage of spermatozoa exhibiting abnormal heads and flagella. In *Ccdc13^-/-^* spermatozoa, predominant defects included abnormal sperm heads with abnormal tails and normal sperm heads with abnormal tails. Compared to *Pcm1^+/+^* spermatozoa, *Pcm1^-/-^* spermatozoa showed a significantly higher percentage of abnormal head morphology, with 18.24±6.20% exhibiting defects, whereas only 6.79±1.77% of *Pcm1^+/+^* spermatozoa displayed similar abnormalities (Figure 4E-F).

TEM analysis of sperm ultrastructure showed that WT and *Pcm1^-/-^* spermatozoa possessed well-defined mitochondrial sheaths in the midpiece, fibrous sheaths along the principal piece, outer dense fibers in the midpiece and proximal principal piece, and a typical “9 + 2” axoneme (Figure 4G-H). In contrast, *Ccdc13^-/-^* spermatozoa exhibited severe disorganization of the axoneme and all accessory structures (Figure 4G). Additionally, *Ccdc13^-/-^* spermatozoa showed the absence or disruption of the central pair structure, along with splitting among several double microtubule doublets (Figure 4G, red asterisk). Thus, knockout of *Ccdc13* causes severe sperm flagellar malformation, leading to male infertility. Contrarily, knockout of *Pcm1* only results in asthenospermia and partial deformity of the sperm head without affecting the formation of the sperm flagellum.

### Flagellum biogenesis in spermiogenesis depends on CCDC13 but not PCM1

Spermatogenesis is a complex and continuous biological process. The seminiferous epithelium cycle is divided into 12 stages, which can be identified by PAS staining of testicular sections 52. The process from acrosome-forming cells to mature spermatids is referred to as spermiogenesis, which involves 16 distinct morphological stages 53. We conducted a stage-wise examination of the morphology of the seminiferous epithelium in *Ccdc13^-/-^* and *Pcm1^-/-^* mice. In testis sections from WT, *Ccdc13^-/-^* and *Pcm1^-/-^*mice, round spermatids differentiated normally into elongating spermatids at stage IX. However, in the testes of *Ccdc13^-/-^* and *Pcm1^-/-^* mice, abnormal elongated spermatids were observed at stages X-XI (Figure S3A-B, red circles). PAS staining further revealed irregular, club-like heads in spermatids at later stages (steps 11-16) in both *Ccdc13^-/-^* and *Pcm1^+/+^* mice (Figure S4A-B). These findings suggest defects in the function of the manchette, a transient microtubular structure involved in sperm head shaping during spermatid elongation. Immunofluorescence staining with α/β-tubulin antibodies revealed that manchette formation in spermatids was normal in steps 8-10 for both *Ccdc13^-/-^* and *Pcm1^-/-^* mice. However, in steps 11-12, the manchettes were abnormally elongated compared to those in WT mice (Figure 4I-J).

**Figure 5:**
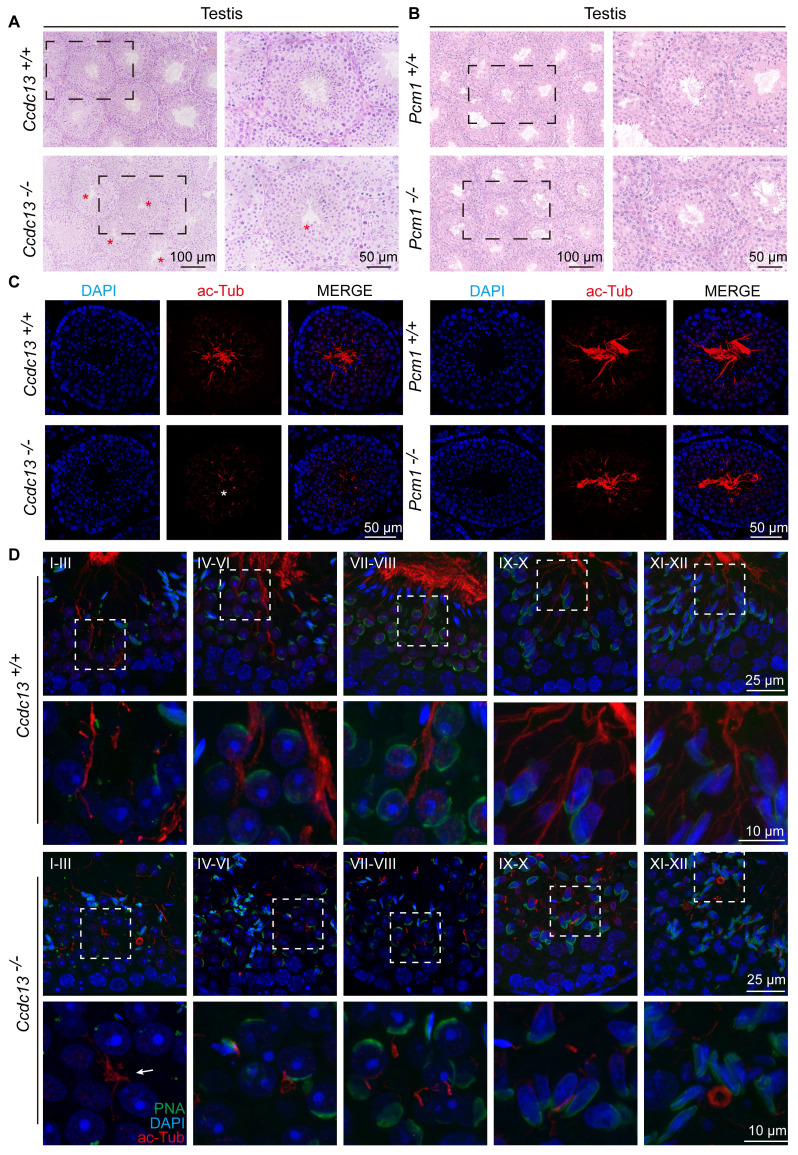
** Sperm flagellum biogenesis is disrupted in *Ccdc13^-/-^* mice, but not in *Pcm1^-/-^* mice.** (A and B) H&E staining of the seminiferous tubules of *Ccdc13^+/+^*, *Ccdc13^-/-^*, *Pcm1^+/+^* and *Pcm1^-/-^* male mice. Red asterisks indicate flagella defects. (C) Immunofluorescence of anti- acetylated tubulin (red) antibodies in testicular sections from *Ccdc13^+/+^*, *Ccdc13^-/-^*, *Pcm1^+/+^* and *Pcm1^-/-^* male mice. The white asterisk indicates flagella defects. (D) Comparison of flagellum biogenesis in testicular sections from *Ccdc13^+/+^* and *Ccdc13^-/-^* mice across different stages. Sperm flagella were stained with anti-acetylated tubulin (red), the acrosome was stained with PNA (green). Enlarged images (indicated by dashed boxes) are displayed in the lower panels. The white arrow indicates that the flagellar axonemes in step 1-3 spermatids of *Ccdc13^-/-^* mice fail to properly elongate.

Further examination of the seminiferous tubules using H&E staining showed tubular lumens in both WT and *Pcm1^-/-^* mice. Flagella were visible as developing spermatids underwent maturation (Figure 5A-B). In contrast, no well-formed flagella were observed in the seminiferous tubules of *Ccdc13^-/-^* mice (Figure 5A, red asterisks). Immunofluorescence using an anti-acetylated tubulin (ac-Tub) antibody confirmed this finding, showing a marked reduction in flagellar axonemes in *Ccdc13^-/-^* mice (Figure 5C, white asterisk), indicative of impaired flagellum formation. To investigate the cause of sperm flagellum defects in *Ccdc13^-/-^* mice, we examined flagellar development using immunofluorescence analysis with the anti-ac-Tub antibody and peanut agglutinin (PNA) to identify which stages were affected by *Ccdc13* knockout. Flagellar axoneme elongation begins at steps 2-3 of early spermiogenesis, originating from the distal centriole 50. In *Ccdc13^-/-^* mice, flagellar axonemes in step 1-3 spermatids failed to elongate properly, unlike the well-defined flagella observed in *Ccdc13^+/+^* mice (Figure 5D, white arrow). Altogether, these results indicate that CCDC13 is critical for proper flagellar axoneme elongation during the early stages of spermatid development, whereas PCM1 is not essential for this process.

### CCDC13 interacts with IMT, IFT-associated proteins, and flagellar components during flagellum biogenesis

**Figure 6:**
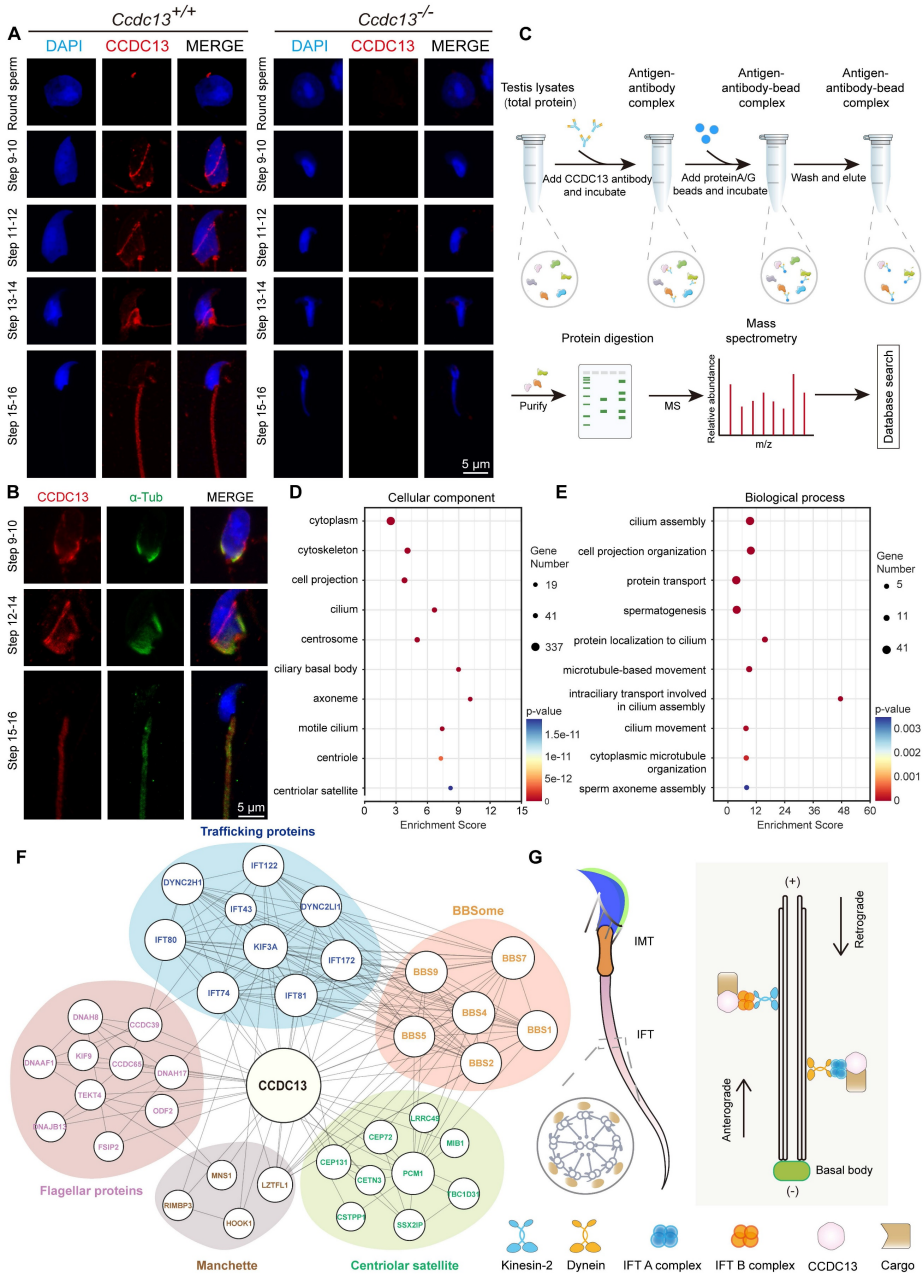
** CCDC13 interacts with IMT and IFT-associated proteins and flagellar components to participate in sperm flagellum biogenesis.** (A) Testicular germ cells were isolated from the testes of *Ccdc13^+/+^* and *Ccdc13^-/-^* adult mice, followed by immunofluorescence staining using antibodies against CCDC13 (red) and DAPI to counterstain the nucleus (blue). (B) Immunofluorescence staining of α-tubulin (green) and CCDC113 (red) in developing germ cells. The manchette and sperm tail were stained with the anti-α-tubulin antibody. (C) A schematic of the experimental workflow depicting the identification of CCDC13-interacting proteins in the testis via immunoprecipitation (IP) and subsequent mass spectrometry analysis. (D and E) GO-term enrichment analysis of CCDC13-interacting proteins was performed using DAVID (Database for Annotation, Visualization and Integrated Discovery), revealing a significant enrichment of terms related to centriolar and axonemal structures, as well as spermatogenesis and sperm axoneme assembly. (F) PPI network between CCDC13 and its interactors include flagellar proteins, trafficking proteins, manchette proteins, components of the BBSome complex, and satellite proteins. The size of the circle in the PPI network reflects the degree of interaction between the protein and other proteins. (G) The schematic diagram illustrates that CCDC13 is involved in flagellar protein transport during sperm flagellum biogenesis.

To further investigate the role of CCDC13 during spermiogenesis, we performed immunofluorescence analysis of CCDC13 in spermatids at various developmental stages. Punctate CCDC13 signals were observed near the nuclei of round spermatids, and its localization was detected at the skirt-like structure surrounding the spermatid head from steps 9 to 14, as well as at the testicular sperm neck and flagellum. Notably, around steps 15 to 16, CCDC13 progressively shifted towards the flagellum of the spermatid (Figure 6A). We next examined the localization of CCDC13 in elongating and elongated spermatids to assess its association with microtubular structures. Co-staining with antibodies against CCDC13 and α-tubulin (a marker for the manchette and flagellum) demonstrated the presence of CCDC13 along the manchette in elongating spermatids, as well as its colocalization with α-tubulin in the flagellum of elongated spermatids (Figure 6B). To investigate the mechanisms underlying the regulation of sperm flagellum biogenesis by CCDC13, we performed immunoprecipitation (IP)-mass spectrometry using the CCDC13 antibody in testis lysates to explore the CCDC13 interactome (Figure 6C).

GO term enrichment analysis of CCDC13-interacting proteins, carried out using DAVID (Database for Annotation, Visualization and Integrated Discovery) 54,55, revealed significant enrichment in terms related to centriolar and axonemal structures, as well as processes involved in protein transport, spermatogenesis, and sperm axoneme assembly (Figure 6D-E, Table S5). To further investigate the function of PCM1 in spermatogenesis, we performed IP-mass spectrometry using the PCM1 antibody in testis lysates to identify interacting partners. GO term enrichment analysis revealed that PCM1-interacting proteins were significantly enriched in sperm flagellum-associated proteins and were involved in sperm flagellar motility and cilium-dependent cell motility (Figure S5A-B, Table S5).

IMT and IFT, the two main cargo transport systems, play critical roles in sperm flagellum biogenesis. Both systems share similar cytoskeletal components, including actin-containing microfilaments and microtubules, which serve as “tracks” to transport cargo proteins. Flagellar structural and motor proteins are transported along sperm manchette microtubules to the base of the flagellum, and subsequently to the developing flagellum via IFT 56,57. Both IMT and IFT enable bidirectional movement within multicomponent transport systems, facilitated by molecular motors such as kinesin-2 and dynein-2 and driving the transport of cargo within IFT protein-containing rafts. Specifically, KIF3A, the motor subunit of kinesin-2, acts as the anterograde motor to move the IFT B complex during sperm tail development, while DYNC2H1 and DYNC2LI1, subunits of dynein, function as retrograde motors to transport the IFT A complex 58. The BBSome complex, composed of eight BBS proteins (1, 2, 4, 5, 7, 8, 9, 18), serves as a membrane coat complex. It interacts with proteins involved in intraflagellar transport, playing a key role in cilia formation and regulating the trafficking of receptors and other transmembrane proteins within the cilium 59. Notably, our analysis of the CCDC13 interactome in the testis revealed a close association between CCDC13 and flagellar and trafficking proteins. These included transport motors and IFT scaffolding proteins, which are components of the BBSome complex and satellite proteins (Figure 6F). These findings strongly indicate that CCDC13 is essential for flagellar protein transport during sperm flagellum biogenesis (Figure 6G).

### In CCDC13-null spermatids, defective transport of HOOK1 and ODF2 coincided with abnormal sperm flagellum development

**Figure 7:**
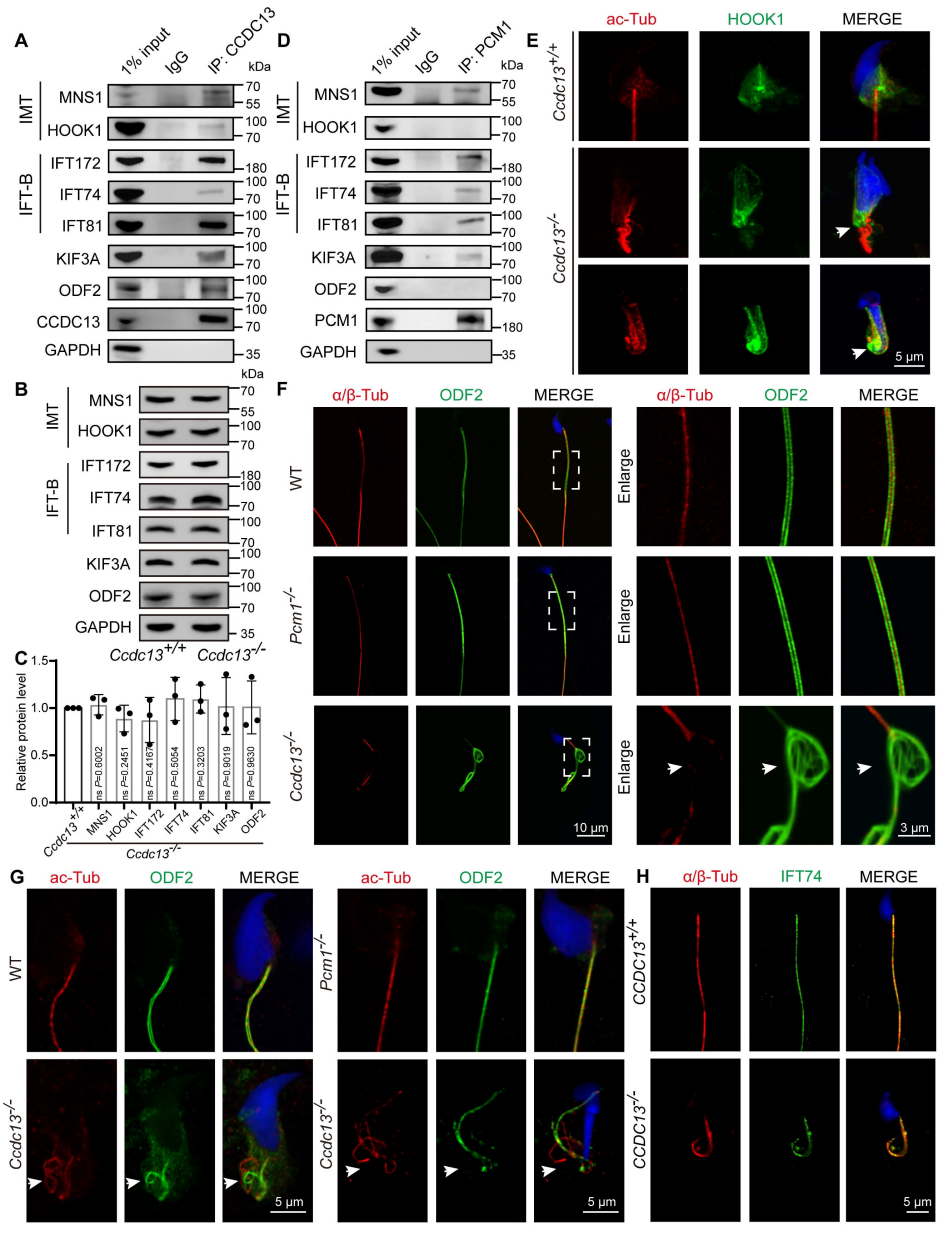
** CCDC13 deficiency disrupts the transport of HOOK1 in IMT and ODF2 in IFT.** (A) Co-IP assays confirmed the interactions between endogenous CCDC13 and intramanchette transport (IMT)-associated proteins—MNS1 and HOOK1—as well as intraflagellar transport (IFT) B components—IFT172, IFT74, and IFT81—along with KIF3A and ODF2 in mouse testes. (B) Western blots show similar protein levels of MNS1, HOOK1, IFT172, IFT74, IFT81, KIF3A and ODF2 in lysates from *Ccdc13^+/+^* and *Ccdc13^-/-^* mouse testes. GAPDH served as a loading control. (C) Quantification of the relative protein levels (n = 3 independent experiments). Data are presented as the mean ± SD. ns: indicates no difference. (D) The interactions between PCM1 and these proteins were also examined by co-IP. PCM1 interacted with MNS1, IFT172, IFT74, IFT81 and KIF3A, but not with HOOK1, or ODF2. (E) Immunostaining images of elongating spermatids from *Ccdc13^+/+^* and *Ccdc13^-/-^* mice labeled with antibodies against HOOK1 (green) and acetylated tubulin (red). DAPI stains nuclei (blue). White arrows indicate that HOOK1 is abnormally located in the caudal part of the manchette. (F) Immunofluorescence staining with antibodies against ODF2 (green) and α/β-tubulin (red) in epididymal spermatozoa from *Ccdc13^+/+^* and *Ccdc13^-/-^* mice. DAPI stains nuclei (blue). White arrows indicate the severely disturbed axoneme and ODF structure. (G) Immunostaining images of elongating spermatids from *Ccdc13^+/+^* and *Ccdc13^-/-^* mice labeled with antibodies against ODF2 (green) and acetylated tubulin (red). DAPI stains nuclei (blue). White arrows indicate defective transport of outer dense fibers along the axoneme. (H) Immunofluorescence staining with antibodies against IFT74 (green) and α/β-tubulin (red) in epididymal spermatozoa from *Ccdc13^+/+^* and *Ccdc13^-/-^* mice. DAPI stains nuclei (blue).

To determine whether CCDC13 deficiency affects IMT and IFT transport processes, we characterized the interactions between CCDC13 and IMT- or IFT-related proteins. *In vivo* co-immunoprecipitation (co-IP) assays confirmed that CCDC13 interacts with IMT-associated proteins (MNS1 and HOOK1), IFT B components (IFT172, IFT74, and IFT81), as well as the flagellar trafficking protein KIF3A and the flagellar component ODF2 (Figure 7A). Importantly, CCDC13 deficiency did not affect the protein levels of these interactors (Figure 7B-C). We also examined the endogenous interactions of PCM1 with these proteins in testicular tissue and found that PCM1 interacts with MNS1, IFT172, IFT74, IFT81, and KIF3A, but not with HOOK1 or ODF2 (Figure 7D). HOOK1 is a manchette-associated protein that facilitates cargo transport to the axoneme in elongating spermatids by binding to motor proteins 60, 61. To examine the effect of CCDC13 deficiency on cargo trafficking, we conducted immunofluorescence staining in *Ccdc13^+/+^* and *Ccdc13^-/-^* elongating spermatids, co-staining with anti-HOOK1 and anti-acetylated tubulin (ac-Tub), which mark the manchette and the flagellum. In *Ccdc13^+/+^* elongating spermatids, HOOK1 localized to the neck region and along the manchette, whereas in *Ccdc13^-/-^* spermatids, HOOK1 abnormally accumulated in the caudal part of the manchette, coinciding with abnormal sperm flagellum development (Figure 7E, white arrows). These results suggest that CCDC13 is crucial for intramanchette transport to the axoneme during sperm flagellum biogenesis.

ODF2 is a major protein of the sperm flagellar outer dense fibers. It is critical for the structural integrity of the sperm flagellum that surrounds the “9 + 2” axoneme in the mid and principal pieces, and defects in ODF2 transport in the flagellum are associated with MMAF 62-64. To further investigate the functional relationship between CCDC13 and ODF2, we conducted immunofluorescence staining on mature spermatozoa from WT, *Pcm1^-/-^*, and *Ccdc13^-/-^* mice. Anti-α/β-tubulin antibody was used to label the sperm flagellar axoneme, while anti-ODF2 antibody was used to visualize outer dense fibers. In *Ccdc13^-/-^* spermatozoa, consistent with transmission electron microscopy results showing severe disorganization of the axoneme and outer dense fibers (Figure 4G), we observed significant malformations in the α/β-tubulin and ODF2 signals, which were less organized compared to those in WT and *Pcm1^-/-^* spermatozoa (Figure 7F). Given that CCDC13 interacts with ODF2 *in vivo*, we hypothesized that CCDC13 regulates ODF2 transport along the axoneme to facilitate flagellar formation.

To test this hypothesis, we examined the distribution of ODF2 along the axoneme in developing spermatids from WT, *Pcm1^-/-^*, and* Ccdc13^-/-^* mice. Immunofluorescence analysis revealed that in *Ccdc13^-/-^* spermatids, ODF2 signals were not properly localized around the axoneme (Figure 7G, white arrows), indicating that ODF2 transport in the flagellum was severely disturbed. To investigate whether CCDC13 deficiency impacts IFT, we examined the localization of IFT74, a core component of the IFT-B complex, in mature spermatozoa from *Ccdc13^+/+^* and *Ccdc13^-/-^* mice. Despite abnormalities in the sperm flagellum of *Ccdc13^-/-^* mice, the IFT74 signal remained intact (Figure 7H). Collectively, these data demonstrate that CCDC13 cooperates with IMT and IFT-associated proteins, as well as flagellar components, to regulate HOOK1 transport in the IMT process and ODF2 transport in the IFT process, thus playing a critical role in sperm flagellum biogenesis.

## Discussion

Research on centriolar satellites has primarily focused on elucidating their composition, proteomes, and regulation of centrosomal and ciliary functions 12, 13, 16, 19-21. However, their tissue-specific functions, particularly in regulation of sperm flagellum function in the testis, have not been systematically explored. In this study, we first generated expression heatmaps of 66 satellite components across human and mouse tissues and found that most of the satellite components expressed in the testis are associated with ciliary functions. We then compared the evolutionary patterns of ciliary-related centriolar satellite proteins across flagellated species. The comparison enabled us to classify these proteins based on their conservation and to identify core conserved satellite proteins that are critical for sperm flagellum biogenesis. Our findings underscore the complexity of satellite protein evolution and highlight the distinct protein requirements for flagellum biogenesis across species.

In our analysis, we classified ciliary-related satellite components into four groups based on their distribution patterns across flagellated and non-flagellated species. Group I includes six satellite proteins (BBS4, DZIP1, CCDC113, CCDC13, CEP131, and TEX9), which are consistently present in flagellated species, suggesting they may play fundamental roles in flagellum biogenesis. Group II consists of satellite protein homologues that are present in some flagellated species but absent in non-flagellated species, suggesting that these proteins may contribute to flagellum biogenesis but are not universally required. In contrast, the centriolar satellite scaffolding component PCM1, along with other proteins from Group III, is less conserved across flagellated species and is primarily found in vertebrates, suggesting that these proteins may not be conserved for flagellum biogenesis. Finally, Group IV comprises satellite proteins conserved in both flagellated and non-flagellated species, yet not directly associated with ciliogenesis, suggesting their functions are more diverse and not related to cilia or flagellum formation. Thus, our evolutionary analysis provides valuable insights into the roles of centriolar satellites in flagellum biogenesis for future research.

Centrioles serve as nucleation sites for cilia, with microtubules extending to form the axoneme of the cilium or flagellum. This role in axoneme formation is conserved during spermiogenesis. The centriole pair attaches to the cell membrane and initiates the formation of the axoneme, which subsequently differentiates into the sperm flagellum 65. We hypothesize that satellite proteins present across flagellated species play conserved roles in sperm flagellum biogenesis. In support of this hypothesis, we, together with other groups, have previously validated the roles of BBS4 31, DZIP1 39, 40, CCDC113 45, CEP131 35, and TEX9 43 in sperm flagellum biogenesis. These proteins form Group I, which are the most conserved proteins in flagellated species. We also compared the roles of CCDC13, a member of Group I, with those of PCM1, a less-conserved protein in Group III. In mice, our findings revealed that *Ccdc13* knockout in the C57BL/6J background led to pup death within 10 days after birth. Pups showed ependymal ciliogenesis abnormalities, leading to severe hydrocephalus. Similarly, recent studies using epiblast-derived *Ccdc13* conditional knockout mice have shown that CCDC13 deficiency leads to significantly enlarged lateral ventricles, indicative of congenital hydrocephalus 66. Knock-in GFP reporter analysis from this group further revealed strong CCDC13 expression in the choroid plexus and ependyma 66, highlighting the importance of CCDC13 in these processes. Our solution to the pup lethality problem was to cross C57BL/6J with ICR mice, providing live *Ccdc13*-knockout pups with ~30% surviving for more than 2 months. Surviving male mice were infertile due to severe flagellar defects, accompanied by abnormal nuclei.

PCM1 functions as a key centriolar satellite scaffolding protein and has been implicated in various ciliary disorders 17, 21, 49, 67. Studies in human *PCM1^-/-^* retinal pigment epithelial cells, mouse inner medullary collecting duct (IMCD3) cells, and mouse multiciliated ependymal cells have demonstrated that PCM1 is essential for ciliogenesis, as its deficiency leads to reduced cilium assembly 47-49. However, its function in flagellum biogenesis remains less understood. Unlike primary or motile cilia, the sperm flagellum exhibits a distinct structural organization and regulatory mechanisms. In our study, *Pcm1* knockout mice were generated by a large-fragment deletion of exons 3-6 using CRISPR/Cas9, which resulted in a frameshift and truncated protein on a C57BL/6N background. These mice exhibited asthenospermia and partial sperm head deformities, but showed no defects in sperm flagellum biogenesis. A recent study using gene trap insertion into intron 4 to generate *Pcm1^-/-^* mice on a C57BL/6J background reported ventricular enlargement, progressive defects in neuronal cilia maintenance, and late-onset behavioral abnormalities. However, no perinatal lethality or early phenotypes associated with ciliary dysfunction were observed 68. Conversely, another study employed CRISPR/Cas9-mediated genome editing to generate two *Pcm1*-deficient mouse lines, although the genetic background was not specified. In line 1, exon 2 was targeted, with a 10-bp deletion at c.5-14 causing a frameshift and resulting in a truncated protein. In line 2, a 5-bp deletion at c.796-800 in exon 6 similarly caused a frameshift and truncated protein. These mice exhibited over 50% perinatal lethality, and the surviving animals displayed severe phenotypes including hydrocephalus, cerebellar hypoplasia, oligospermia, and partially penetrant hydronephrosis 48.

The phenotypic discrepancies observed across these three *Pcm1* knockout models may be attributed to differences in mouse genetic backgrounds and the targeted genomic regions. Such findings underscore the complexity of genotype-phenotype correlations, wherein mutations in the same gene can lead to divergent biological outcomes depending on genetic context. Indeed, genetic background effects have been documented for numerous disease-associated mutations 69. For example, the clinical heterogeneity associated with the Ala467Thr mutation in the mitochondrial polymerase-γ (*POLG*) gene illustrates how identical homozygous mutations can give rise to a wide range of phenotypes, from Alpers-Huttenlocher syndrome to peripheral neuropathy and ophthalmoplegia 70. Similarly, associations between *USP26* mutations and male infertility have been found to be significant in Asian populations but not in European or American populations 71. Among the identified variants, the c.1737G>A mutation appears to contribute to male infertility, whereas c.576G>A and c.1090C>T do not show significant associations 72. Notably, despite phenotypic variability, all three *Pcm1* knockout models consistently demonstrate that PCM1 is dispensable for flagellum biogenesis. Our study further revealed that PCM1-interacting proteins are significantly enriched in sperm flagellum-associated components, particularly those involved in flagellar motility and cilium-dependent cell movement. These findings suggest that PCM1 may contribute to the proper localization of proteins essential for sperm motility, highlighting a functional divergence between its roles in ciliary and flagellar systems.

Centriolar satellites exhibit diverse cellular distributions, ranging from clustering at centrosomes and perinuclear regions to scattering throughout the cytoplasm 19, 73. This variability suggests that centriolar satellites may have cell type- and tissue-specific functions, which remain largely unexplored, particularly during spermatogenesis. In our recent and current studies, we observed the localization of CCDC113 45 and CCDC13 at the sperm neck, manchette, and flagella in elongating spermatids, with subsequent translocation to the flagellum in elongated spermatids. The stage-specific redistribution of CCDC113, and CCDC13 suggests that they may function as cargo transported by IMT and IFT to participate in sperm flagellum biogenesis. We further verified that CCDC13 interacts with IMT components (MNS1 and HOOK1), IFT-B components (IFT172, IFT74, and IFT81), and the molecular motor KIF3A in the testis. In the absence of CCDC13, both the localization of HOOK1 in the IMT process and the transport of ODF2 in the IFT process were disrupted. Additionally, we identified several centriolar satellite proteins in the interactome of CCDC13 in testicular cells, including the highly conserved ciliary satellite components BBS4 and CEP131. Deficiency of BBS4 has been reported to impair sperm flagellum biogenesis 31. Deficiency of CEP131, which localizes at the sperm neck in later-stage spermatids, has been shown to disrupt microtubule-based trafficking of both IMT and IFT and to lead to abnormal sperm head morphology and the absence of flagella in *Cep131* mutant spermatids 35. These findings suggest that CCDC13 may cooperate with BBS4 and CEP131 to regulate the assembly and elongation of the sperm flagellum. This underscores the critical role of centriolar satellite proteins in forming a complex network essential for sperm flagellum biogenesis. Furthermore, DZIP1 has been reported to localize at the sperm neck in human spermatozoa, where the sperm centrioles are found. Mutations in *DZIP1* lead to failures in intraflagellar transport and disorganization of centrioles, impairing flagellum formation 40. Collectively, these findings highlight the dynamic localization of highly conserved centriolar satellite components during spermiogenesis and their crucial role in regulating microtubule-based trafficking during flagellum formation.

Taken together, our comparative genomic approach provides insights into the functional categorization of centriolar satellite proteins and identifies a set of highly conserved proteins in flagellated species—BBS4, DZIP1, CCDC113, CCDC13, and CEP131, TEX9—that are essential for sperm flagellum biogenesis. In contrast, the centriolar satellite scaffolding component PCM1 plays an auxiliary role in these processes. The identification of these proteins offers valuable insights into the genetic mechanisms underlying flagellum function, as well as their evolutionary development. Considering the evolutionary conservation of these proteins across a wide range of species, their functions are likely conserved as well. Thus, we speculate that mutations in the human orthologs of these genes could be associated with male infertility.

## Supplementary Material

Supplementary figures S1-S5 and tables S1-S2.

Supplementary table S3.

Supplementary table S4.

Supplementary table S5.

## Abbreviations

CCDC: coiled-coil domain-containing
MMAF: multiple morphological abnormalities of the sperm flagella
IMT: intramanchette transport
IFT: intraflagellar transport
PCR: polymerase chain reaction
WT: wild-type
H&E: hematoxylin-eosin
PAS: Periodic acid-Schiff
PNA: peanut agglutinin
TEM: transmission electron microscopy
MS: mitochondrial sheath
FS: fibrous sheath
ODF: outer dense fibers
DMT: doublet microtubule
CP: central pair
co-IP: co-immunoprecipitation
