# Supplementary material for: A subset of evolutionarily conserved centriolar satellite core components is crucial for sperm flagellum biogenesis
Source: Theranostics. 2025 Jun 12;15(14):7025–44. doi: 10.7150/thno.117118 (PMC12203921; doi:10.7150/thno.117118)
Supplement: Supplementary file 1 — Supplementary figures S1-S5 and tables S1-S2. [file thnov15p7025s1.pdf]

Supplementary Materials for

A subset of evolutionarily conserved centriolar satellite core components is crucial for sperm flagellum biogenesis

This file includes:

Figures S1 to S5
Table S1
Table S2

Other Supplementary Material for this manuscript includes the following:

Tables S3
Expression data for 66 centriolar satellite components in human and mouse tissues available at the National Center for Biotechnology Information.
Tables S4
Identification of protein homologues of 43 ciliary-related centriolar satellite components across 12 species.
Tables S5
CCDC13 and PCM1 interactome in the mouse testis.

Supplementary Figures

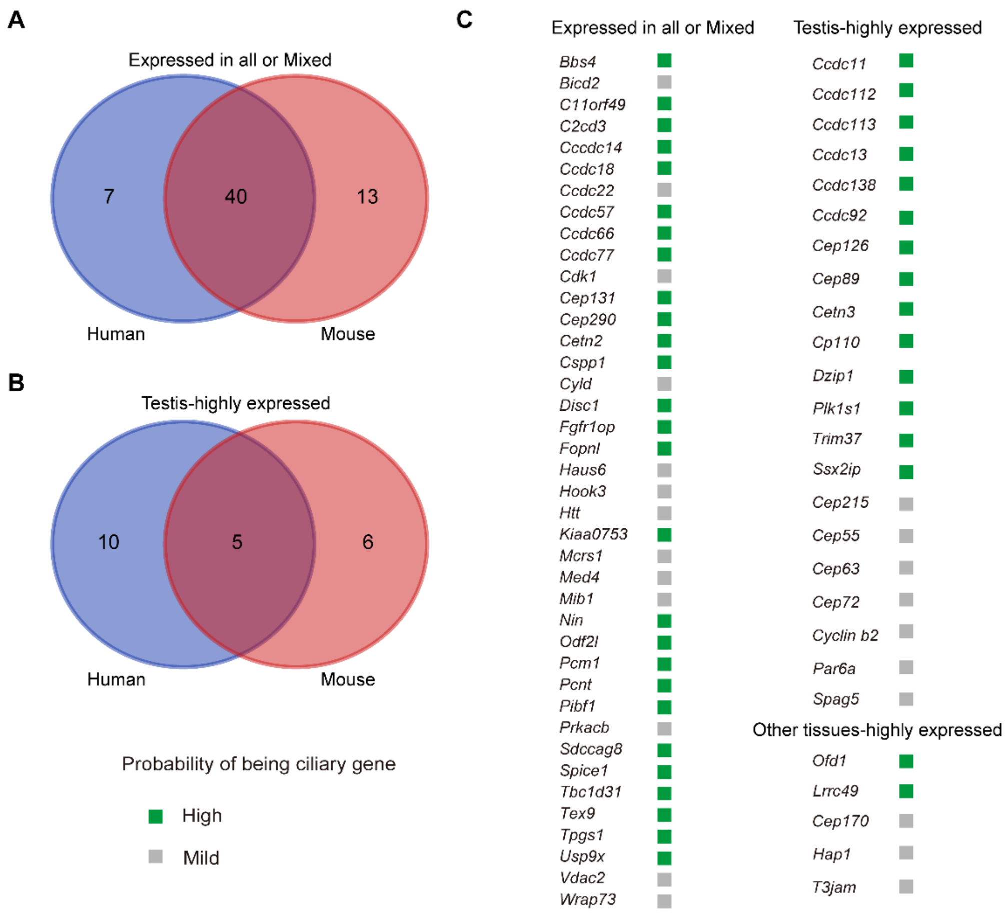

Figure S1. Prediction of 43 ciliary genes in centriolar satellites using the Ciliogenics database.

(A) The Venn diagram compares the satellite components that are "Expressed in All or Mixed" in both human and mouse. (B) The Venn diagram compares the testis-highly expressed satellite components between human and mouse. (C) Prediction of ciliary genes in 66 centriolar satellite components across three categories using the Ciliogenics database.

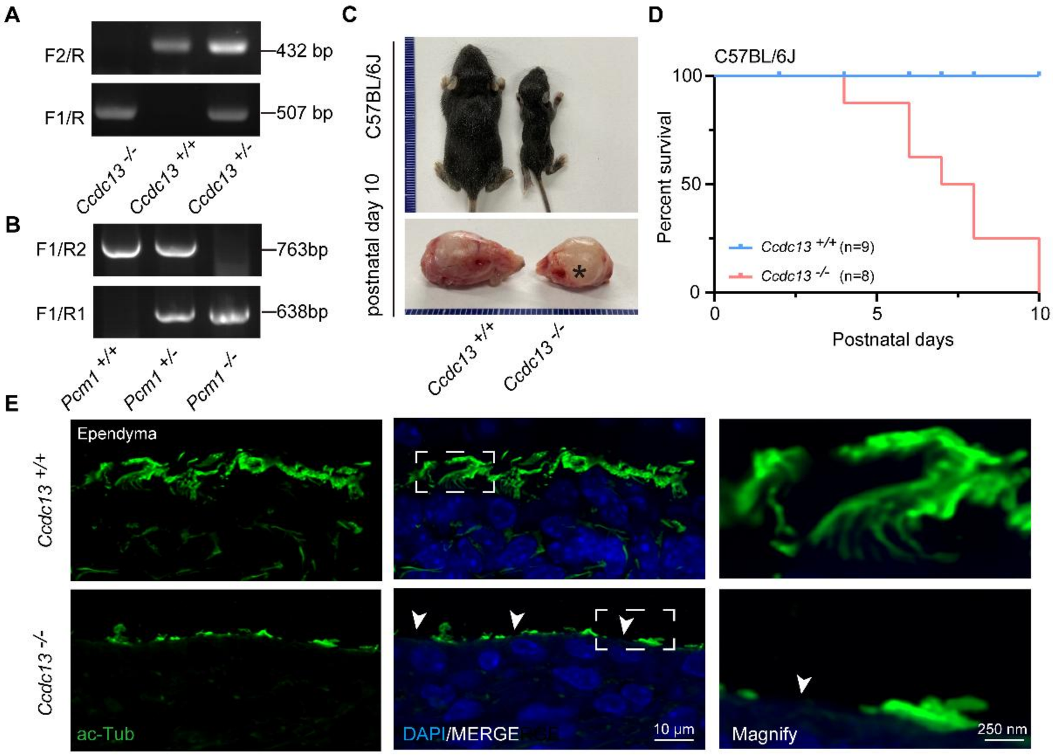

Figure S2. ***Ccdc13*** **knockout leads to hydrocephalus and ependymal ciliogenesis abnormalities.**

(A and B) Genotyping to identify *Ccdc13*- and *Pcm1*-knockout mice. (C) Photograph of *Ccdc13*$^{-/-}$ mice and WT littermate in the C57BL/6J background at postnatal day 10. Black asterisk indicates the hydrocephalus in *Ccdc13*$^{-/-}$ mice. (D) Survival rate of postnatal *Ccdc13*$^{-/-}$ mice in the C57BL/6J background. (E) Immunofluorescence of anti- Acetylated tubulin (green) antibodies in ependyma sections from *Ccdc13*$^{+/+}$ and *Ccdc13*$^{-/-}$mice. The white arrowheads indicate ciliogenesis defects.

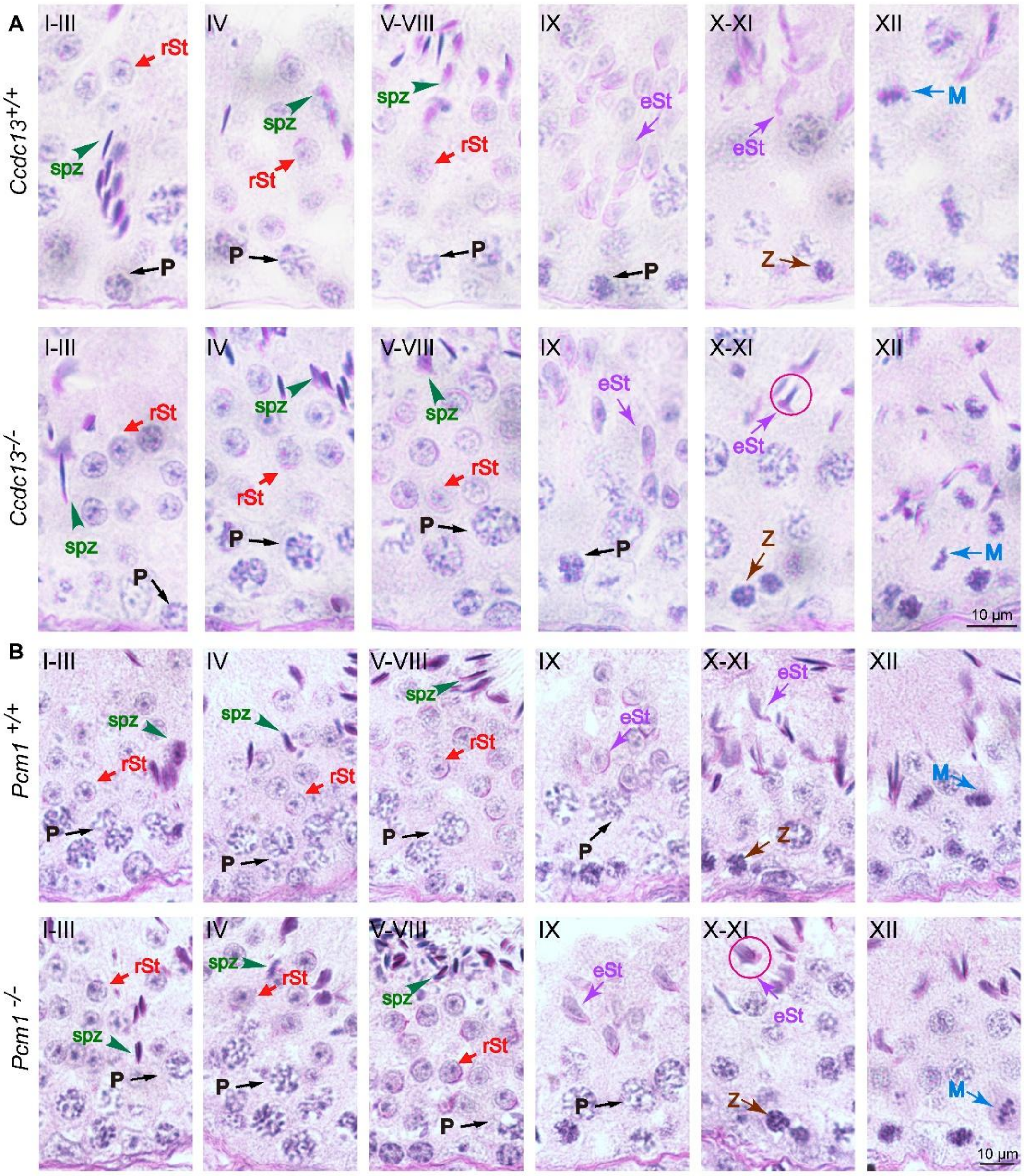

Figure S3. Abnormal spermiogenesis in *Ccdc13*$^{-/-}$ mice and *Pcm1*$^{-/-}$ mice. (A and B) PAS staining of *Ccdc13*$^{-/-}$ and *Pcm1*$^{-/-}$ testis sections showed abnormal sperm nuclear shape. The red circle indicates an abnormal elongated spermatid at stages X-XI in *Ccdc13*$^{+/+}$, *Ccdc13*$^{-/-}$, *Pcm1*$^{+/+}$, and *Pcm1*$^{-/-}$ mice. P, pachytene spermatocyte; Z, zygotene spermatocyte; M, meiotic spermatocyte; rSt, round spermatid; eSt, elongating spermatid; spz: spermatozoa.

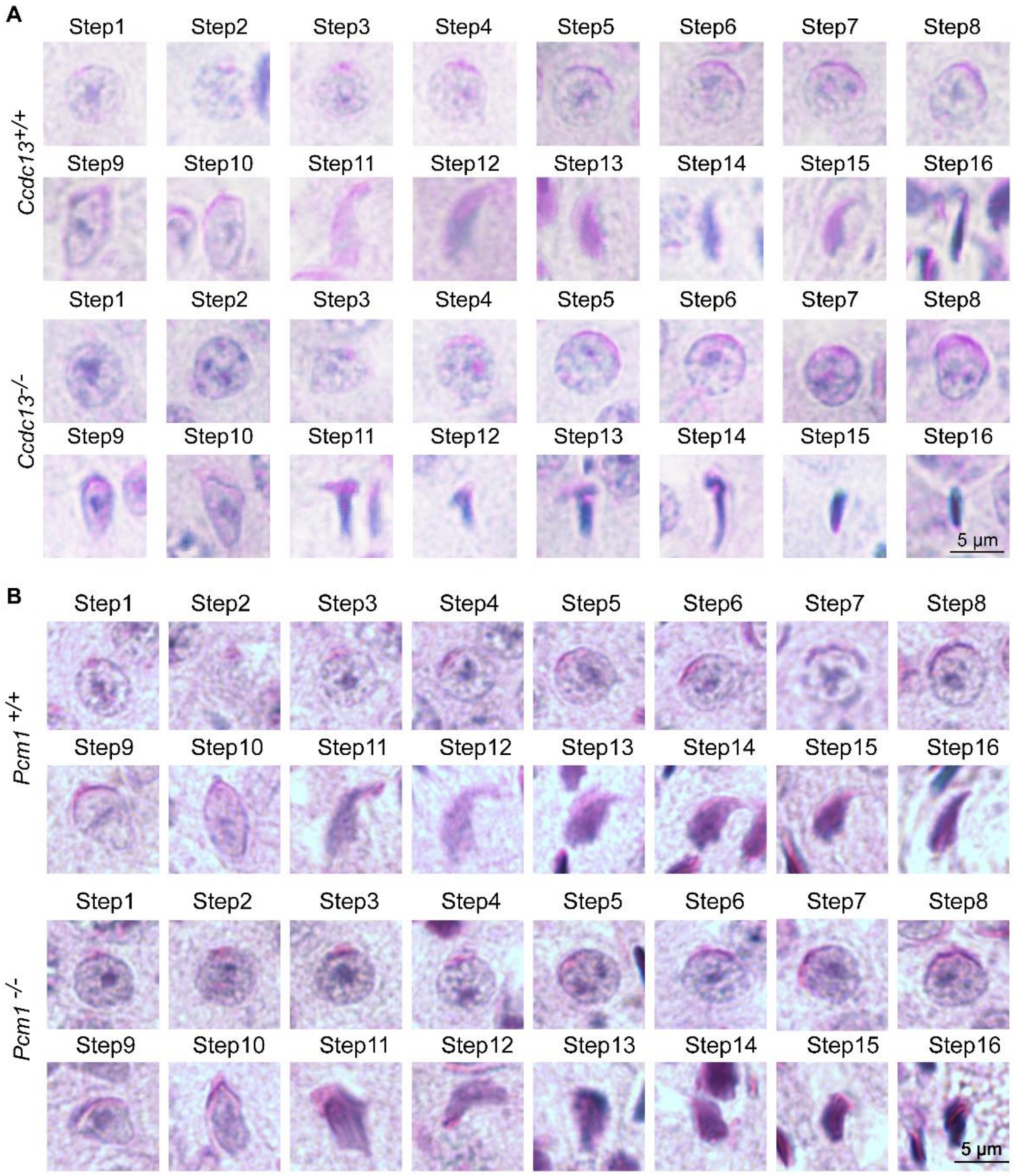

Figure S4. Sperm head malformations in *Ccdc13*$^{-/-}$ mice and *Pcm1*$^{-/-}$ mice.

(A and B) PAS staining of spermatids at different steps from *Ccdc13*$^{+/+}$, *Ccdc13*$^{-/-}$, *Pcm1*$^{+/+}$, and *Pcm1*$^{-/-}$ mice was performed. Abnormal, club-shaped heads were observed in later steps (steps 11–16) spermatids in *Ccdc13*$^{-/-}$ and *Pcm1*$^{-/-}$ mice.

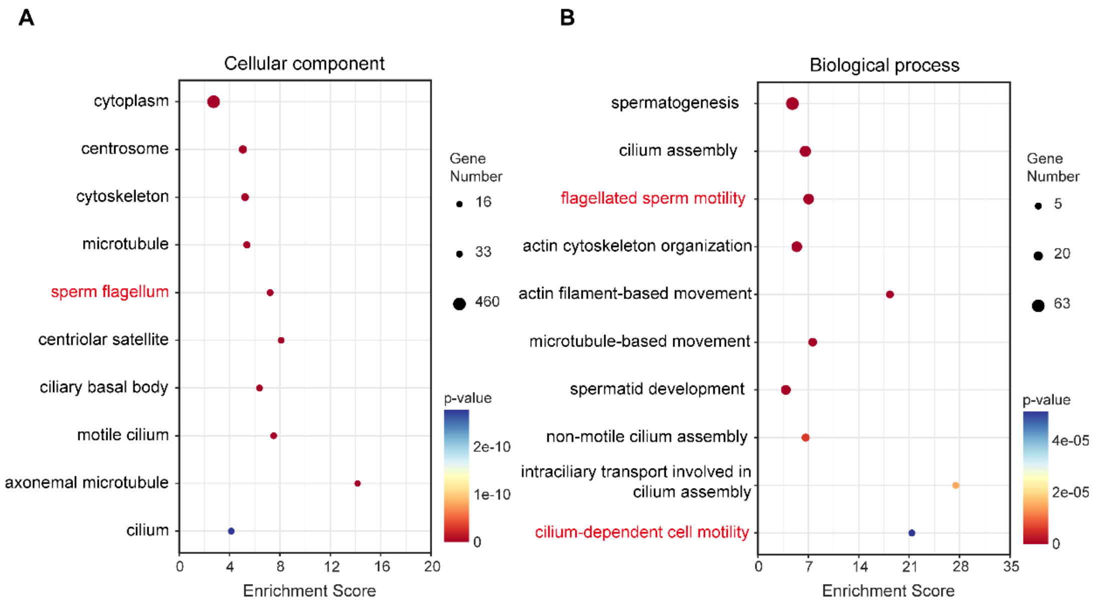

Figure S5. GO term enrichment analysis of PCM1-interacting proteins.

(A and B) GO term enrichment analysis of PCM1-interacting proteins in the testis was performed using DAVID, revealing enrichment in sperm flagellar motility.

Supplementary tables

Table S1. Primers for genotyping *Ccdc13* and *Pcm1* knockout mice.

Primer	Sequence (5'→3')	Product size
Ccdc13 F1	TCCAGGTTTTATGTGAACTTGGCT	KO: 507 bp
Ccdc13 R1	AAGTGTGGCCATCCTACTGACT	
Ccdc13 F2	CAACTCAGTGTCCAGGTGAGCT	WT: 432 bp
Ccdc13 R2	AAGTGTGGCCATCCTACTGACT	
Pcm1 F1	ACTAAAGGAGACCTGTTAAAGACG	KO: 638 bp
Pcm1 R1	TGGCAAACCTAGAGCCCTCC	
Pcm1 F2	ACTAAAGGAGACCTGTTAAAGACG	WT: 763 bp
Pcm1 R2	ACCCTCTTATCACTTGCTACACC	

Table S2. Overview of the antibodies or dyes used in this study.

Overview of the antibodies or dyes used in this study	Manufacturer	Cat No.
CCDC13 (IF, 1:50; WB, 1:1000)	Dia-an Biotech	homemade
PCM1 (WB, 1:1000)	Proteintech	19856-1-AP
α-tubulin antibody (IF, 1:100; WB, 1:5000)	Abclonal	AC012
acetylated tubulin (IF, 1:200)	Sigma-Aldrich	T7451
α/β-tubulin (IF, 1:200)	Abcam	ab44928
IFT74 (IF, 1:50; WB, 1:1000)	Proteintech	27334-1-AP
HOOK1 (IF, 1:50; WB, 1:1000)	Proteintech	10871-1-AP
ODF2 (IF, 1:200; WB, 1:1000)	Proteintech	12058-1-AP
MNS1 (WB, 1:1000)	Proteintech	12693-1-AP
IFT172 (WB, 1:1000)	Proteintech	28441-1-AP
IFT81 (WB, 1:1000)	Proteintech	11744-1-AP
KIF3A (WB, 1:1000)	Proteintech	13930-1-AP
GAPDH (WB, 1:3000)	Proteintech	60004-1-Ig
Alexa Fluor 488 conjugate of lectin PNA (1:200)	Thermo Fisher	L21409
goat anti-mouse FITC (IF, 1:200)	Zhong Shan Jin Qiao	ZF-0312
goat anti-rabbit FITC (IF, 1:200)	Zhong Shan Jin Qiao	ZF-0311
goat anti-mouse TRITC (IF, 1:200)	Zhong Shan Jin Qiao	ZF0313
goat anti-rabbit TRITC (IF, 1:200)	Zhong Shan Jin Qiao	ZF-0316
